# EHMTI-0186. Multi-center 3T MRI study of cortical thickness abnormalities in migraine

**DOI:** 10.1186/1129-2377-15-S1-A4

**Published:** 2014-09-18

**Authors:** S Magon, A May, A Stankewitz, PJ Goadsby, C Schankin, M Ashina, FM Amin, J Müller, CL Seifert, M Chakravarty, T Sprenger

**Affiliations:** 1 Department of Neurology, University Hospital Basel, Basel, Switzerland; 2 Institute for Systems Neuroscience, University of Hamburg, Hamburg, Germany; 3 Headache Group-Department of Neurology, University of California, San Francisco, USA; 4 Danish Headache Center and Department of Neurology Glostrup Hospital, University of Copenhagen, Copenhagen, Denmark; 5 Department of Neurology, Technische Universitaet Muenchen, Muenchen, Germany; 6 Campbell Family Institute CAMH, University of Toronto, Toronto, Canada

## Introduction

A few studies have investigated CTh in patients with migraine showing highly variable and inconsistent results. The relatively small sample sizes of the previous studies and unbalanced study groups may partially explain these inconsistencies.

## Aims

To investigate differences of cortical thickness (CTh) between healthy subjects and migraineurs in a large multicenter MRI study.

## Methods

High-resolution T1-weighted isotropic 3D MRI data acquired at 3 Tesla in 131 patients with migraine (92 patients without aura, 39 with aura; 31±9 years old; 109 women; monthly attack frequency: 3.2± 2.5; disease duration: 14±8.4 years) and 115 matched healthy subjects (29±7 years old; 81 women) were collected from 4 different centers (5 different scanners). CTh was computed using FreeSurfer. In order to investigate group effects, a vertex-wise linear model was used controlling for age, gender and scanner. All statistical analyses were performed using R. False Discovery Rate (p<0.05) was applied to correct for multiple comparisons.

## Results

We found a significantly thinner cortex in patients with migraine as compared to controls in the somatosensory cortex bilaterally, in the left middle frontal gyrus and in the left occipital lobe (V1 and V2). These results were mainly driven by patients without aura. No regions with cortical thickening in patients as compared to controls were found.

## Conclusions

These results confirm abnormalities in specific cortical regions in a large cohort of migraineurs. The areas where we observed reductions of cortical thickness belong to somatosensory, pain and visual networks, all previously implicated in migraine pathophysiology.

